# Condition- and context-dependent variation of sexual dimorphism across lizard populations at different spatial scales

**DOI:** 10.1038/s41598-022-21358-2

**Published:** 2022-10-10

**Authors:** Martina Muraro, Stéphanie Sherpa, Benedetta Barzaghi, Pierluigi Bombi, Danilo Borgatti, Viola Di Canio, Andrea Dalpasso, Mattia Falaschi, Benedetta Gambioli, Raoul Manenti, Silvio Marta, Paolo Momigliano, Veronica Nanni, Claudio Pardo, Elia Lo Parrino, Stefano Scali, Federico Storniolo, Leonardo Vignoli, Marco A. L. Zuffi, Roberto Sacchi, Daniele Salvi, Gentile Francesco Ficetola

**Affiliations:** 1 Department of Environmental Science and Policy, Università Degli Studi Di Milano, Via Celoria 10, 20133 Milan, Italy; 2 Institute of Research On Terrestrial Ecosystems, National Research Council, 00015 Montelibretti, Italy; 3 Department of Sciences, Roma Tre University, 00146 Rome, Italy; 4 Area of Ecology and Biodiversity, School of Biological Sciences, The University of Hong Kong, Pok Fu Lam, Hong Kong; 5 School for Advanced Studies IUSS, Science, Technology and Society Department, 25100 Pavia, Italy; 6 Department of Ecological and Biological Sciences, Tuscia University, 01100 Viterbo, Italy; 7 Museo Di Storia Naturale, C.so Venezia 55, 20121 Milano, Italy; 8 Department of Earth and Environmental Sciences, University of Pavia, 27100 Pavia, Italy; 9 Museo Di Storia Naturale -Università Di Pisa, via Roma 79, 56011 Calci (Pisa), Italy; 10 Department of Health, Life and Environmental Sciences, University of L’Aquila, 67100 L’Aquila, Italy; 11 Laboratoire d’Écologie Alpine (LECA), University Grenoble Alpes, CNRS, University Savoie Mont Blanc, 38000 Grenoble, France

**Keywords:** Evolutionary ecology, Sexual selection, Herpetology

## Abstract

The evolution of sexual dimorphism (SD) is driven by intricate interplays between sexual and natural selection. When it comes to SD variation within populations, however, environmental factors play a major role. Sexually selected traits are expected to be strongly dependent on individual body condition, which is influenced by the local environment that individuals experience. As a consequence, the degree of SD may also depend on resource availability. Here, we investigated the potential drivers of SD expression at two sexually dimorphic morphometric traits, body size (snout vent length) and head shape (head geometric morphometrics), in the Italian wall lizard (*Podarcis siculus)*. We assessed the existence of condition- and context-dependent SD across ten islands of the Aeolian archipelago (southern Italy), at within- and among-population scales. We observed strong geographical variation of SD among islands, and tested three potential SD predictors related to resource availability (individual body condition, ecosystem productivity, temperature). Body condition and ecosystem productivity were the main drivers of body size SD variation, and body condition was also the main driver for head shape SD. Our results highlight that the expression of SD in the Italian wall lizard is both condition- and context-dependent. These results are congruent at within- and among-populations scales highlighting that spatial multi-scale analysis represents a useful approach to understand patterns of SD expression.

## Introduction

Sexual dimorphism (SD) is widespread in the animal world and can involve extensive variation in morphological, physiological and behavioral traits^1^. Understanding the processes leading to SD has been a central topic of evolutionary studies since Darwin’s work^2^; these studies have shown that the evolution of SD is driven by a complex interplay between sexual and natural selection. Understanding the variation of sexually dimorphic traits is complicated by the fact that many traits are dependent on individual condition^3^, which is in turn a product of resource availability and the individual’s efficiency at translating the available resources into fitness^4^. Consequently, sexually dimorphic traits often show strong phenotypic plasticity across gradients of resource availability, and the patterns of variation within and between sexes are caused by both genetic and developmental processes^3^. During organism development, the allocation of resources to sexual traits can be costly and their expression is tightly linked to the availability of resources^1,5,6^. For instance, in *Hyalella* amphipods, male gnathopod size (a sexually selected trait) is more susceptible to resource stress (food availability) than non-sexual or female traits^7^.

Given the costs of sexually dimorphic traits, theory predicts that the expression of traits exaggerated by sexual selection should be strongly dependent on the condition of individuals^3^. As a result, sexual selection acting on male traits should lead to condition-dependent sexual dimorphism, where differences between males and females are stronger in individuals in better condition, and males’ traits are more strongly affected by variation in conditions than female traits^3^. Assessing the conditions experienced by individuals in natural populations can be challenging, but measures such as the body condition index (BCI, obtained from the residuals of the relationship between body mass and body length), can provide a good estimate of the overall foraging success and fitness of individuals^8^. The expression of SD can thus correlate with BCI, as evidenced for the sex-specific coloration (yellow cheek-parches) at population level in the Hermann’s tortoise^3,9^.

The individual condition of animals is strongly affected by the environment, hence the degree of SD is also expected to depend on resource availability (context-dependent SD)^10^. Several environmental features can be used as proxies of resource availability or can determine variation in fitness-related traits, thus triggering variation of sexual dimorphism. Environmental variables that can affect SD include ecosystem productivity (e.g.^11^) and temperature, the latter having particularly strong impacts on the physiology, morphology, behavior and metabolism of ectotherms^12,14,15^. The effect of environmental features on SD can be assessed at different spatial scales. Some studies focused on differences between individuals within population, while others used an eco-geographical approach, evaluating broad scale drivers of differences among populations^10,15–17^. While both scales can provide useful information on the potential drivers of SD, multi-scale studies are required to assess whether the processes determining the variation in SD between individuals within a population are the same across spatially isolated populations.

Lizards are a good model for studying the degree of SD because they show strong variation in direction and magnitude of SD at multiple traits, across and within species^18^. The family Lacertidae generally shows a male-biased SD, with larger male body size and head dimensions driven by sexual selection (combat and mating performances), while females usually have larger abdomen length (a trait related to fecundity)^19,20^. Such considerable SD is also found in the Italian wall lizard, *Podarcis siculus*^21^. This species, widespread in the Mediterranean basin^22,23^, has a broad ecological tolerance and shows variation of SD across populations^16^.

The aim of this study is to test whether resource availability determines variation of SD within and among populations of *P. siculus* on the Aeolian archipelago (Southern Italy). To this end we measured SD in body size and head shape, and tested if the expression of SD at these traits shows the same response to environmental variation within and across islands^16,24^. We evaluated the effect of three predictor variables representing resource availability on the degree of SD: (i) individual body condition (BCI) to investigate condition-dependent SD, (ii) ecosystem productivity (estimated through the Normalized Difference Vegetation Index, NDVI) and (iii) land surface temperature, that may affect context-dependent SD not accounted for by BCI. We predict that SD should be more pronounced in individuals showing better body conditions, and/or in environments with more resources (higher productivity or warmer temperature). Furthermore, we assessed the effects of these predictors at two spatial scales: the individual scale (male–female differences within populations) and the island scale (SD degree among spatially isolated populations), to evaluate whether scaling issues affect the detection of the drivers of SD.

## Results

**Figure 1:**
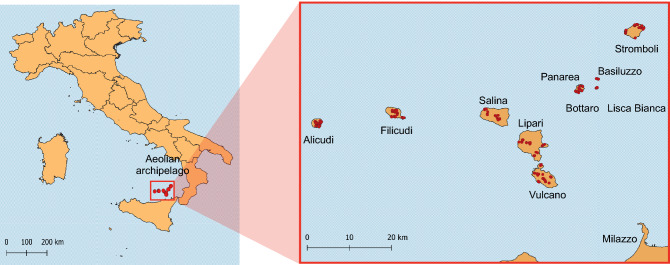
Study area in the Aeolian archipelago and sampling locations of the 408 sampled individuals (red dots). Lisca Bianca and Bottaro islets are geographically close and points are partially overlapped. The map was created using QGIS^68^.

We captured 408 adult lizards (239 males and 169 females) from the seven main islands of the Aeolian archipelago (Alicudi, Filicudi, Salina, Panarea, Stromboli, Lipari, Vulcano) and from three islets around Panarea (Bottaro, Lisca Bianca, Basiluzzo) (Fig. 1). The number of individuals per island ranged between 48 and 59 in large islands and between 7 and 12 in the islets (Table S1). Snout-vent length (SVL) was significantly different across islands and between sexes, males being longer (68.34 ± 9.69 mm) than females (58.82 ± 9.71 mm) (ANOVA: effect of sex: F_1, 397_ = 225.32, *p* < 0.001; island: F_9, 397_ = 4.12, *p* < 0.001; Fig. S1a).

**Figure 2:**
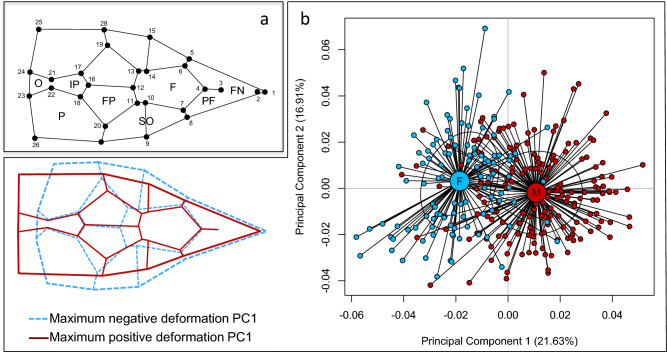
Head shape sexual dimorphism. (**a**) Localization of the 28 landmarks recorded on the head of *Podarcis siculus*. Solid black lines: mean shape among all individuals. Nomenclature of scales: F: frontal, FN: frontonasal, FP: frontoparietal, IP: interparietal, N: nasal, O: occipital, P: parietal, PF, prefrontal, SO: supraocular. (**b**) Principal Component Analysis (PCA) on Procrustes shape coordinates, with 38% of total variance accounted by the first two PCs. Schematic representation of landmark deformations on the first PC reconstructed from grids and vectors (exaggeration factor of 2). Solid red lines: male maximum deformation, dashed blue lines: female maximum deformation.

Head shape variation (landmark-based geometric morphometrics, Fig. 2a) was assessed using individual scores (PC) of a principal component analysis on head shape coordinates of a subset of 302 individuals (Table S1). Since we were interested in head shape dimorphism, we tested the effect of sex on each PC using linear mixed effects models (LMMs), including island of origin as random factor and body size as covariate. PC1 showed strong differentiation between sexes (F_1, 294.02_ = 64.24, *p* < 0.001, marginal R^2^ = 0.54, conditional R^2^ = 0.61), while differences between sexes for PC2 were very small (F_1, 292.08_ = 0.866, *p* = 0.35; marginal R^2^ = 0.04, conditional R^2^ = 0.44, Fig. 2b). We therefore focused analyses of head shape dimorphism on PC1. Head shape variation on PC1 accounted for 21.6% of total variation (Fig. 2b). The two sexes differed mainly in the shape of the area around the back of the head, with males having longer parietal scales compared to females (Fig. 2b). We found significant variation of head shape between sexes and across the ten islands (ANOVA: effect of sex: F_1, 290_ = 230.09, *p* < 0.001; effect of island: F_9, 290_ = 5.07, *p* < 0.001; Fig. S1b).

### Individual scale (within populations)

**Figure 3:**
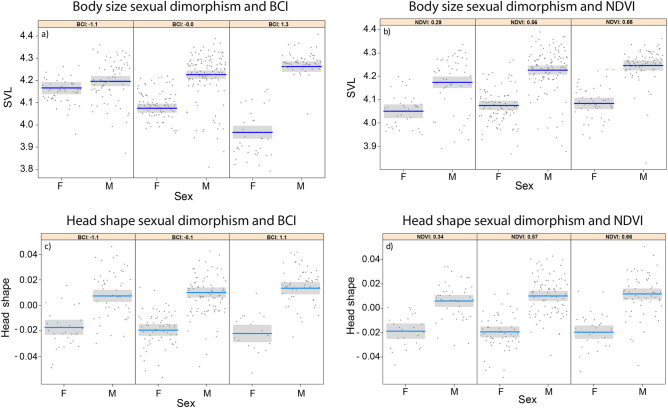
Conditional plots showing the relationship between sexual dimorphism and the environmental drivers included in the best AICc models at the individual scale (N = 408). Phenotypic traits: (**a**, **b**) SVL, (**c**, **d**) head shape; drivers: interactions between (**a**, **c**) sex and BCI and (**b**, **d**) sex and NDVI. Male–female differences are divided in three categories of environmental drivers: low (10th quantile), intermediate (median), and high (90th quantile) of BCI (**a**, **c**) and NDVI (**b**, **d**).

We assessed the effect of environmental variables (BCI, NDVI, temperature) on body size and head shape differences between males and females at the individual scale using LMMs, testing the significance of interactions between sex and each environmental variable and including island as a random factor to take into account the non-independence of lizards collected on the same island. The best-AICc model explaining the SVL of individuals included BCI, NDVI, sex, the interaction between sex and BCI, and the interaction between sex and NDVI. Males were consistently larger than females (F_1, 401.4_ = 8.34, *p* = 0.004; Fig. 3). A strong effect of the interaction between sex and BCI showed that body size scaled differently with body condition in each sex (F_1, 404.8_ = 121.56, *p* < 0.001; Fig. 3a). Furthermore, lizards from localities with high NDVI were larger (F_1, 382.6_ = 19.64, *p* < 0.001), and the difference between males and females slightly increased in sites with high NDVI, even though the effect of the interaction between sex and NDVI was not significant at the 0.05 level (F_1, 401_ = 2.87, *p* = 0.09; Fig. 3b) (Table 1).

**Table 1:** Best models assessing the effect of environmental predictors on sexual dimorphism (SD) at individual and island scales. The dependent variables of models are: SVL; head shape; body size SD and head shape SD. Models are ranked according to their AICc values. Only models with *w* > 0.02 and with AICc lower than the null model are shown (see Table S2). The sign of the relationship is in parentheses.

Dependent	Predictors	AICc	∆ AICc	*w*	*R*^2^_M_	*R*^2^_C_
**Individual scale**						
SVL	BCI (-), NDVI ( +), Sex, Sex*BCI, Sex*NDVI	2561.6	–	> 0.999	0.51	0.55
Head shape	BCI (-), NDVI ( +), Sex, Sex*BCI, Sex*NDVI	− 1618.6	–	0.36	0.44	0.49
	BCI (-), Sex, Sex*BCI	− 1618.4	0.2	0.33	0.42	0.49
	NDVI ( +), Temp ( +), Sex, Sex*NDVI, Sex*Temp	− 1617.4	1.1	0.2	0.44	0.48
**Island scale**						
Body size SD	BCI ( +), NDVI ( +)	− 30.9	–	0.45	0.68	
	BCI ( +)	− 30.8	0.1	0.43	0.48	
	NDVI ( +)	− 28.2	2.7	0.12	0.34	
Head shape SD	BCI ( +)	− 67.6	–	> 0.999	0.43	

∆AICc: difference between the AICc of a model and the best AICc; *w*: Akaike’s weight of the model; *R*^2^_M_: marginal *R*2; *R*^2^_C_: conditional *R*^2^.

For head shape, AICc identified three models showing similar AICc values (Table 1). All models suggested that head shape was different between males and females (F_1, 294.7_ = 5.654, *p* = 0.018 in the best-AICc model). Furthermore, the model with the lowest AICc value suggested that head shape differences between males and females were stronger in individuals with better body condition (F_1, 294.6_ = 4.32, *p* = 0.038; Fig. 3c), and in individuals found in sites with high NDVI, although the latter effect was not significant (F_1, 294.2_ = 1.943, *p* = 0.164; Fig. 3d). Head shape was unrelated to the NDVI of the location where individuals were captured, nor to the BCI of individuals (in both cases, *p* > 0.3). However, for head shape, there was uncertainty in model selection. The competing models included two simpler models, the first model included neither NDVI nor the interaction between NDVI and sex, while the second model, which showed a slightly higher AICc, included neither BCI nor the interaction between sex and BCI, but included NDVI, soil temperature, and the respective interactions (Table 1). All the results remained consistent after the removal of the islets with the smallest sample size (Table S3a).

### Island scale (among populations)

**Figure 4:**
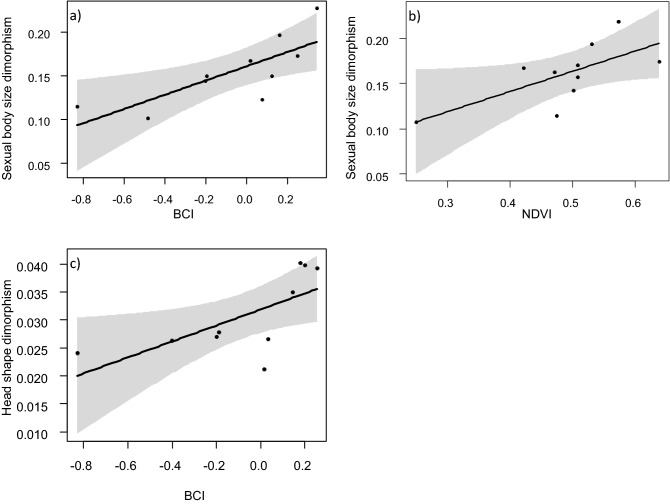
Conditional plots showing the relationship between sexual dimorphism and the environmental drivers included in the best AICc models at the island scale (N = 10). Sexual dimorphism: (**a**, **b**) body size dimorphism, (**c**) head shape dimorphism; drivers: (**a**-**c**) BCI, (**b**) NDVI. Black line: regression line, grey shaded area: 95% confidence interval.

The effect of environmental variables on body size SD (Lovich and Gibbons index) and head shape SD (average Euclidean distance among all male–female pairs on PC1 shape variable) at the island scale was assessed using linear models (LMs), testing the significance of each environmental variable averaged among all the sampling points of each island. The best-AICc model suggested that body size dimorphism is higher in islands where the average BCI is largest (F_1, 7_ = 13.5, *p* = 0.008; Fig. 4a) and with highest NDVI (F_1, 7_ = 5.87, *p* = 0.046; Fig. 4b). Simpler models, only including one of these variables, showed slightly higher AICc values (Table 1). For head shape dimorphism, the best-AICc model included the average BCI of the island, indicating that sexual dimorphism is higher in islands where individuals have better BCI (F_1, 8_ = 6.684, *p* = 0.032; Fig. 4c) (Table 1). All the results remained consistent after the removal of the islets with the smallest sample size (Table S3b).

## Discussion

The degree of sexual dimorphism can be influenced by individual condition and environmental resource availability^3,10^. By assessing the drivers of SD at both the individual and island scales (Table 1), we demonstrate that (i) the difference in body size and head shape between males and females in Italian wall lizards varies across individuals and islands, and (ii) the pattern of variation of SD is consistent at different geographical scales corresponding to within- and among-population levels. Despite the limited number of sampled islands, our results all support the hypothesis that body condition and context-dependent factors (i.e. ecosystem productivity) concur to drive SD degree in *P. siculus*.

Sexual dimorphism is widespread in lizards, body size and head shape being the main dimorphic characters^20,24,25^. In *P. siculus* both of these characters strongly differ between sexes. Sexual dimorphism of these traits is related to two behaviors widespread in lacertid lizards: male-male fights and forced copulation^20^. Head shape is an extremely good predictor of bite force, which in lizards is fundamental for territory defense, female accession and copula^26^.

Several studies demonstrated geographic variation of sex-related traits, with significant relationships with island features (e.g.^10,27^). Morphological changes can occur rapidly on islands, providing different contexts of resource availability and environmental features^28–30^. We show that body size and head shape in *P. siculus* strongly vary across the different islands of the Aeolian archipelago. The variation across islands can be the result of phenotypic plasticity and/or local adaptation^31^, and multiple processes can contribute to this variation. Sexual selection generally favors large male size, and the extent of differences between sexes can be affected by environmental features, for instance because when more resources are available phenotypic plasticity allows maximal divergence of growth trajectories. Furthermore, we cannot exclude that variation of resources determines variation of sexual and / or natural selection, for instance if more resources relax natural selection. Distinguishing between these hypotheses is challenging, and long-term common rearing environments would be required to fully tease apart the role of these processes. Nevertheless, the positive relationship between SD and resource availability was clearly observed across individuals within the population, supporting the hypothesis of an important role of phenotypic plasticity^32–34^.

The incredible diversity of sexually selected traits has been of particular interest to evolutionary biologists. However, the intraspecific variation in these traits often remains unexplained^35^ and is rarely investigated at multiple spatial scales. Our analyses show that differences between males and females are related to both the condition of individuals and the resource availability in the environment at the individual and island scales^3^, body condition having the strongest effect on the expression of sexual dimorphism. Populations with the largest average BCI values also showed the strongest SD (Fig. 4 a-c). Furthermore, within-population differences between males and females were exacerbated for the individuals with the highest BCI (Fig. 3), and this condition-related pattern of SD expression was confirmed in different traits (body size and head shape). Males in better condition can allocate more resources to sexually selected traits; which are costly and are tightly linked with body condition. For example, in the crowned leafnose snake, better body condition of males determines longer tails, which are important for mating^36^.

Nevertheless, body condition alone is not enough to fully explain the variation of SD. The individual condition is strongly affected by the context in which individuals live and, therefore, to the availability of resources^1,5,6^, and BCI variation is unable to fully capture the environmental variation experienced by individuals. Among the environmental features that can be used as a proxy of resource availability in the wild and determine variation of SD, we found support for a role of ecosystem productivity (here measured on the basis of NDVI, which is a proxy of plant productivity and / or biomass^37,38^) at two different spatial scales. Primary productivity strongly determines the amount of available resources and often affects size SD expression. For instance, the positive relationship between size SD and primary productivity in the Iberian newt (body size) and in tidal-marsh sparrows (bill size) suggests that abundant productivity may relax natural selection^11,39^. The two morphometric traits studied here do not follow the same context-dependent pattern of SD expression, as the positive relationship between ecosystem productivity and SD was only observed for body size but not head shape SD. It is possible that size SD shows a stronger plastic response in relation to resource availability compared to head shape. Indeed, several studies have shown that head shape may vary with other environmental features such as altitude, urbanization, island area and food niche breadth^40–42^. The size of an island is an alternative proxy for resource availability as well as for population size, because larger islands offer more resources than small ones and populations in large islands experience less demographic stochasticity. Island size has therefore also been related to variation in SD^10,43,44^. Island area and ecosystem productivity are collinear in our system (see method), and we focused on productivity because of its more direct link with the resources available for resources. The strongest importance of productivity is also supported by our data, as alternative models considering island area instead of productivity did not show any significant relationship between island area and degree of SD (Tab S4).

Temperature played a minor role on SD expression compared to body condition and NDVI. This result partially contrasts previous studies. Indeed, temperature plays a crucial role in the fitness of ectotherms, affecting organism physiology, morphology, behavior and metabolism (e.g.^12,45^), and can determine the intensity of sexual selection and the degree of SD^14^. For example, the SD of a seed beetle population decreases in extreme temperature conditions compared to an intermediate temperature in common garden experiments, suggesting a better allocation of resources when individuals experience an optimal thermal environment^13^. In our study system, the variation of thermal conditions across the study area was strong. In the morning, average surface temperature during the warmest semester of the year ranged from 21 °C (mostly at high elevations and in north-facing slopes) to 43 °C. Despite this substantial variation, no population experienced very cold conditions that could strongly limit lizard activity. This suggests that, under the relatively benign conditions of the study area, the high thermoregulation capacity of lizards may allow to buffer environmental variation.

**Figure 5:**
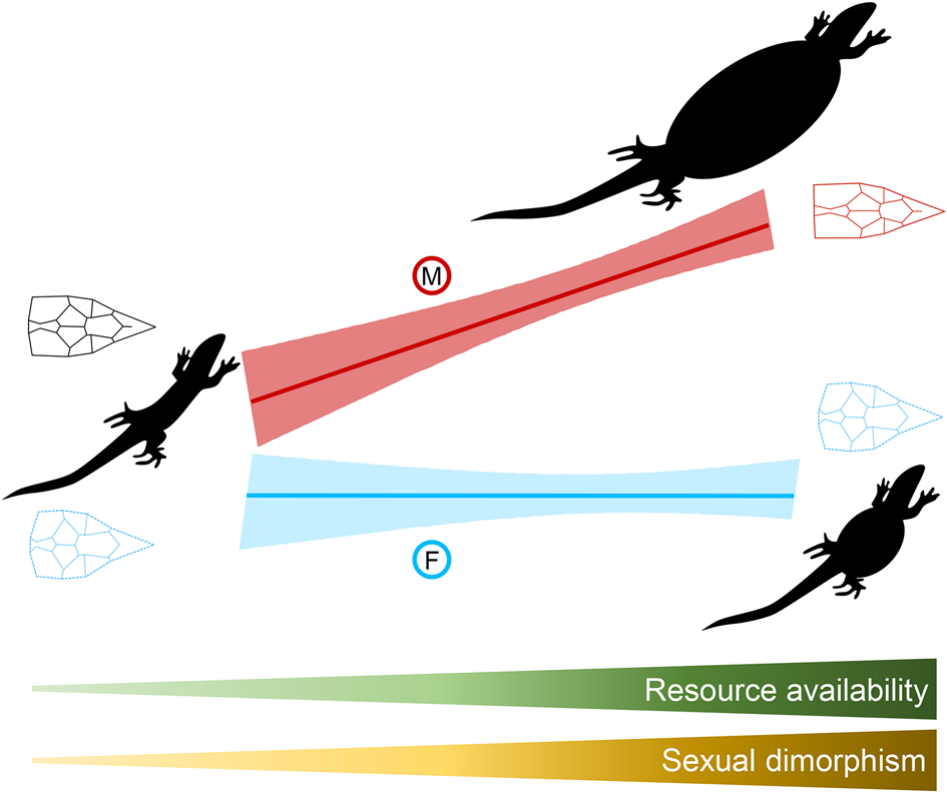
Condition and context-dependent expression of sexual dimorphism in *Podarcis siculus*. General trends of body size and head shape expression according to resource availability in males (M, red) and females (F, blue).

In conclusion, our results highlight that better body condition and higher primary productivity, two proxies for resource availability, can increase the expression of SD. The expression of SD in the Italian wall lizard is both condition- and context-dependent, with context-dependent effects being mostly evident for body size. Abundant accessible resources can determine strong phenotypic plasticity, but might also relax natural selection and lead to increased selection for sexual traits^11,39^. In high-resource habitats, males have longer head parietal scales and body size compared to low-resource habitats, while female head shape and body size do not change in relation to resource availability (Fig. 5), possibly because they invest more in other traits not measured here, e.g. interlimb length^20^. It is also possible that the strength of sexual selection is similar across islands, but variation in energy availability determines different potential for males to achieve the largest body sizes. In other words, variation in environmental conditions can determine a broad range of sexual dimorphism even if sexual selection remains constant. The agreement between our results and what already observed for different species in other geographic areas suggests that spatial variation of SD could be a general pattern for lizard populations facing environmental stressors in resource-constrained habitats^10^. Nevertheless, the joint evaluation of direct measures of individual condition and habitat resource availability will allow a better identification of mechanisms that affecting sexual dimorphism at multiple traits.

## Material and methods

### Data collection

The Aeolian archipelago is composed of seven main islands and several islets, and includes active, dormant and extinct volcanoes. The island volcanic landforms, characterized by altitude from the sea level up to 962 m above sea level (a.s.l), provide a variety of climatic conditions and a high environmental heterogeneity^46^. In September 2021, we sampled lizards from the seven main islands of the Aeolian archipelago (Alicudi, Filicudi, Salina, Panarea, Stromboli, Lipari, Vulcano) and from three islets (Bottaro, Lisca Bianca and Basiluzzo) (Fig. 1). Sampling design covered the whole altitudinal range of each island. Lizards were sexed, measured, and weighed (accuracy: 0.1 g). The head of each individual was photographed in dorsal view using Olympus TG-5 or TG-6 cameras in a photo light box. The pictures were used to perform head geometric morphometrics. We also recorded the GPS coordinates of each captured individual (accuracy: 3 m).

### Phenotypic traits and measures of sexual dimorphism

To analyze sexual dimorphism, we considered three phenotypic traits: 1) snout-vent length (SVL), 2) head size and 3) head shape. SVL was measured with a Vernier caliper^47^ and log-transformed to improve normality. Head size and shape variables were obtained using landmark-based geometric morphometrics. Head geometric morphometrics was performed using 28 landmarks located at intersections and borders of cephalic scales (Fig. 2a; see^19^ for a description of landmarks). Individuals for which any of the landmarks could not be defined properly were excluded from the study. A TPS file with all the individuals was created using tpsUtil version 1.87^48^ and landmarks were digitized using tpsDig2 version 2.31^49^. Variation due to scale, orientation, and position was removed by applying a Procrustes superimposition using the IMP software CoordGen8^50^. We applied a principal component analysis (PCA) on Procrustes-aligned head shape coordinates using the software PCAGen8^51^ and the resulting individual scores on each PC were used as shape variables. Head size was determined as the log-transformed centroid size, but was strongly correlated to SVL (Pearson’s *r* = 0.89, *p* < 0.001, Fig. S2) and was not considered for subsequent analyses.

Sexual dimorphism was thus quantified for body size (SVL) and head shape (scores on PC1). We estimated the size dimorphism with the Lovich and Gibbons index^52^:

$$\mathrm{SDI}=\frac{\mathrm{mean \;SVL \;of \;larger \;sex}\left(\mathrm{males}\right)}{\mathrm{mean\;SVL \;of\; smaller \;sex }\left(\mathrm{females}\right)}-1$$

The head shape dimorphism was calculated as the average of Euclidean distances among all male–female pairs on PC1 shape variable using the usedist R package^53^.

### Potential drivers of sexual dimorphism

We focused on three variables that can represent condition and context variation, and may affect phenotypic traits and sexual dimorphism: body condition of individuals, ecosystem productivity (NDVI), and land surface temperature. Body condition is a fitness-related parameter providing an overall index of animal conditions and foraging success^8^. The body condition index (BCI) was calculated as the residuals from the regression of body mass on SVL. This regression included individuals from both sexes and all the islands, and sex as independent variable to discriminate between males and females. Body mass and SVL were log-transformed (Fig. S3)^54^.

Proxies of productivity and temperatures were obtained from remote-sensing data. As a measure of productivity/peak greenness, we used the Normalized Difference Vegetation Index (NDVI). NDVI is a proxy of photosynthetic activity and green biomass; it can represent resource availability and resource partitioning^37,38^. All the Landsat-8 TOA images (LANDSAT/LC08/C01/T1_TOA—30 m resolution) available for the time frame April 1st—September 30th (year 2015 to 2020) were processed, and the seasonal NDVI maximum, averaged over the years of interest, was calculated. April 1st – September 30th represents the period with highest activity of the study species^55^. Land surface temperatures were retrieved for the same period, following the approach detailed in Ermida et al.^56^. Being ectotherms, lizards are strongly affected by the abiotic environment. Environmental temperature, in particular, affects performance of many biochemical processes and can determine variation at fitness-related traits (e.g. survival rate and fecundity)^57,58^. The Landsat-8 TOA collection was used to retrieve brightness temperature, while the Landsat-8 SR collection (LANDSAT/LC08/C01/T1_SR) for computing fractional vegetation cover (FVC), using standard NDVI thresholds (NDVI_bare_ = 0.2 and NDVI_veg_ = 0.86). Landsat emissivity was obtained by correcting ASTER GEDv3 (NASA/ASTER_GED/AG100_003) surface emissivity for bare ground, using Landsat-8 FVC. Temperature was measured as land surface temperatures. These temperatures were calculated by applying the Statistical Mono-Window algorithm to the Thermal infrared band of Landsat-8 TOA, and implementing information from atmospheric water content (TCWV) from the NCEP/NCAR reanalysis (NCEP_RE/surface_wv). The obtained bi-monthly surface temperatures were averaged over the period April 1st – September 30th. A total of 199 images were used (tile ids: LC08_188033, LC08_188034, LC08_189033), all collected between 09:34 and 09:42 am. All the analyses of satellite imagery were run using the cloud service Google Earth Engine (GEE) and the R package *rgee*^59^.

Island area can be a further measure of resource productivity in insular environments, as larger islands often have more resources^10^. The area of each island was obtained from the ReptIsland database (^60^, available at https://doi.org/10.6084/m9.figshare.14346416). However, island area was strongly correlated with the NDVI averaged across all the sampled points of each island (Pearson’s *r* = 0.90, *p* < 0.001; Fig. S2), thus island area was not considered in further analyses. Differences in SD were unrelated to geographic distances between islands (Mantel’s tests, 9999 permutations; SDI: *r* = − 0.099, *P* = 0.646; head shape dimorphism: *r* = − 0.068, *P* = 0.608).

### Statistical analyses

The analysis of sexual dimorphism was repeated at the i) individual and ii) island scales. We first tested the differences between sexes in body size and head shape among islands using analysis of variance (ANOVA). Then, we built a series of linear mixed effects models (LMMs) (individual scale) and linear models (LMs) (island scale) testing the effect of resource availability (BCI, NDVI, temperature) on body size and head shape dimorphism. At the individual scale, the effect of BCI, NDVI and temperature on body size and head shape was tested as the statistical interaction between sex (M/F) and each predictor^3^. Interactions between sex and predictors account for different responses between males and females to that variables. For individual-scale analyses, we used NDVI and soil temperature of the capture location of each individual and the BCI of each individual. For island-scale analyses, these three variables were averaged among all the sampling points of each island. We included islands as a random factor to consider the non-independence of lizards collected on the same island.

We built models with all the potential combinations of predictors, and calculated the corrected Akaike’s Information Criterion (AICc) for each model. The model with the lowest AICc value was considered to be the “best model”^61^ (see Table S2). AICc can select excessively complex models, consequently we considered a complex model as a candidate model only when it had a lower AICc than the AICc of all its simpler nested models^62^. Moreover, we only considered models with AICc values lower than the null model. For each model, we calculated the AIC weight, which represents the support of the model, given the data and the set of candidate models. Finally, we determined the evidence ratios E = *w*_*i*_*/w*_*j*_ to compare the relative support of the models^63^. We used the marginal and conditional *R*^2^ (*R*^2^_M_ and *R*^2^_C_, respectively), as measures of the variation explained by each model^64^. Three islets (Basiluzzo, Bottaro and Lisca Bianca) had smaller sample size than larger islands. To confirm the robustness of our conclusions, we re-run the best-AICc models after removing these three islets.

We used the lme4, lmerTest, car and MuMIn packages for ANOVA, LLMs and LMs^65^ and visreg package^66^ to produce conditional regression plots. All statistical analyses were performed in R 3.4.2 (R Core Team^67^, http://www.r-project.org).

### Ethics statement

Capture and manipulation of lizards, and all the experimental protocols were authorized by the Italian Ministry for the Environment (prot. 0,037,921.13–04-2021). Lizards were collected by noosing and immediately released in the site where they had been captured after measurements, as specified in the permits of the Ministry for the Environment. All methods were carried out in accordance with relevant guidelines and regulations.

## Supplementary Information

Supplementary Information 1.

Supplementary Information 2.

Supplementary Information 3.

## Data Availability

The datasets supporting the conclusions of this article are included within the article (Supplementary Tables 1 and 2). Geometric morphometrics data are in the form of landmark Procrustes coordinates but digitized pictures are available from the authors.
