# Supplementary material for: Condition- and context-dependent variation of sexual dimorphism across lizard populations at different spatial scales
Source: Sci Rep. 2022 Oct 10;12:16969. doi: 10.1038/s41598-022-21358-2 (PMC9550790; doi:10.1038/s41598-022-21358-2)
Supplement: Supplementary file 3 — Supplementary Information 3. [file 41598_2022_21358_MOESM3_ESM.pdf]

Supplementary material

Manuscript title: Condition- and context-dependent variation of sexual dimorphism across lizard populations at different spatial scales

Martina Muraro[1], Stéphanie Sherpa[1,*], Benedetta Barzaghi[1], Pierluigi Bombi[2], Danilo Borgatti[1], Viola Di Canio[1], Andrea Dalpasso[1], Mattia Falaschi[1], Benedetta Gambioli[3], Raoul Manenti[1], Silvio Marta[1], Paolo Momigliano[4], Veronica Nanni[5], Claudio Pardo[6], Elia Lo Parrino[1], Stefano Scali[7], Federico Storniolo[8], Leonardo Vignoli[3], Marco A. L. Zuffi[9], Roberto Sacchi[8], Daniele Salvi[10], Gentile Francesco Ficetola[1,11]

[1] Department of Environmental Science and Policy, Università degli Studi di Milano, Via Celoria 10, 20133 Milan, Italy.

[2] Institute of Research on Terrestrial Ecosystems, National Research Council, 00015 Montelibretti, Italy

[3] Department of Sciences, Roma Tre University, 00146 Rome, Italy

[4] Department of Biochemistry, Genetics, and Immunology, Universidade de Vigo, 36310 Vigo, Spain

[5] School for Advanced Studies IUSS, Science, Technology and Society Department, I-25100 Pavia, Italy

[6] Department of Ecological and Biological Sciences, Tuscia University, 01100 Viterbo, Italy

[7] Museo di Storia Naturale, C.so Venezia 55, I-20121 Milano, Italy

[8] Department of Earth and environmental Sciences, University of Pavia, 27100 Pavia, Italy

[9] Museo di Storia Naturale -Università di Pisa, via Roma 79, 56011 Calci (Pisa), Italy

[10] Department of Health, Life and Environmental Sciences, University of L'Aquila, 67100 L'Aquila, Italy

[11] Univ. Grenoble Alpes, CNRS, Univ. Savoie Mont Blanc, Laboratoire d'Écologie Alpine (LECA), F-38000 Grenoble, France.

[*] Corresponding author: Stéphanie Sherpa, email: stephanie.sherpa@hotmail.fr

a)

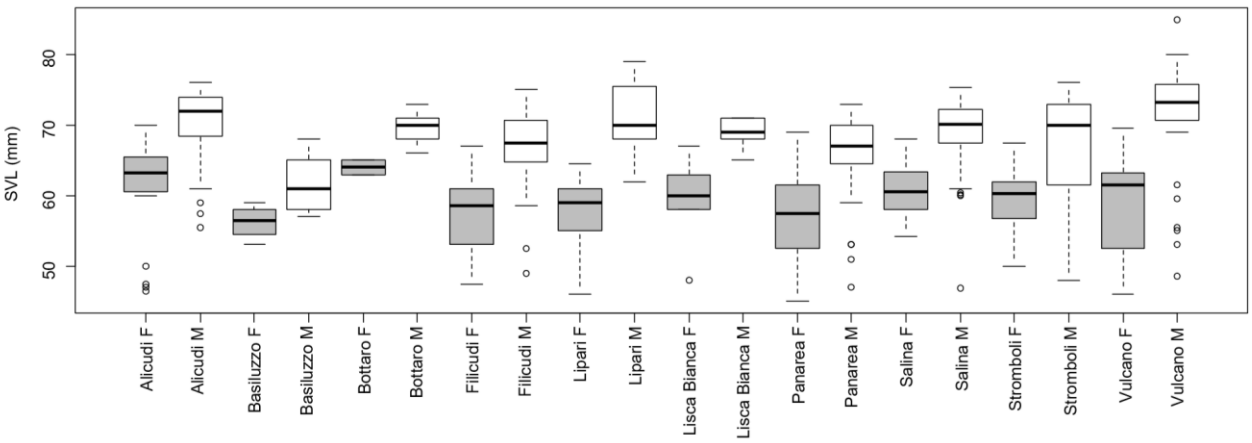

b)

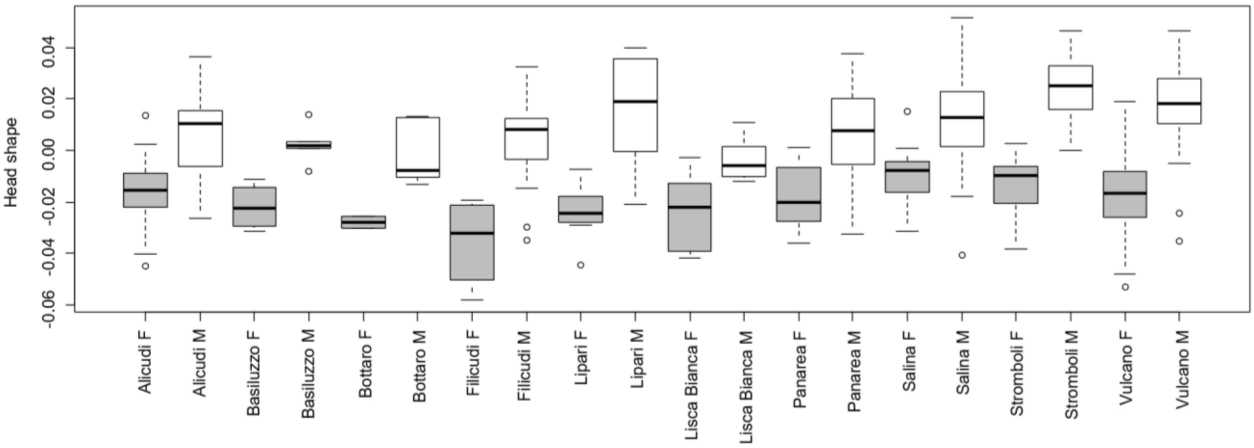

Figure S1. Variation of phenotypic traits of *Podarcis siculus* among the 10 islands: a) SVL, b) head shape. Female (grey) and males (white) are shown separately.

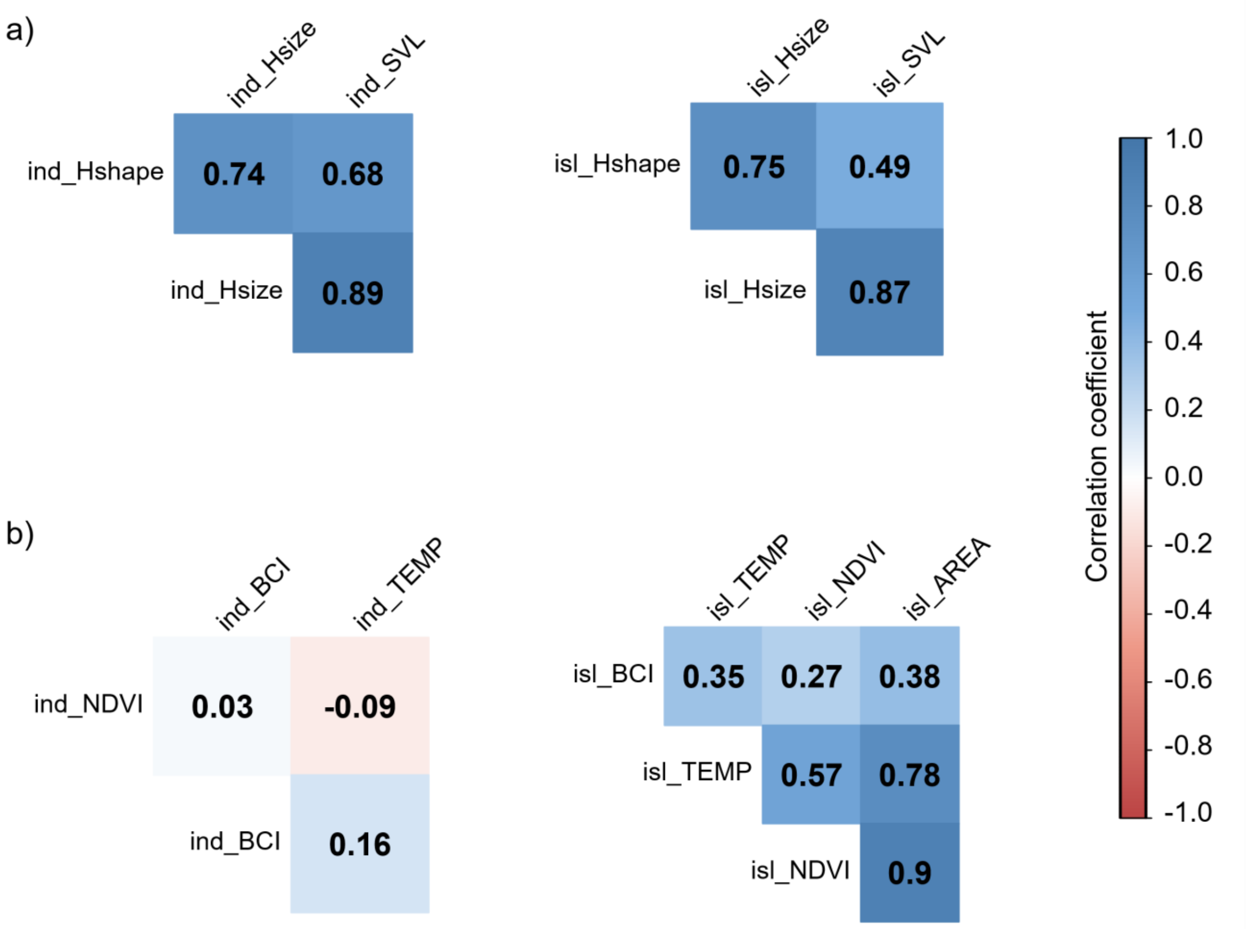

Figure S2. Pearson's correlation coefficients at different scales (ind= individual scale, isl= island scale) for: a) phenotypic traits (SVL, Hsize=head size, Hshape= head shape), b) resource variables (BCI= Body Condition Index, NDVI= Normalized Difference Vegetation Index, TEMP=temperature).

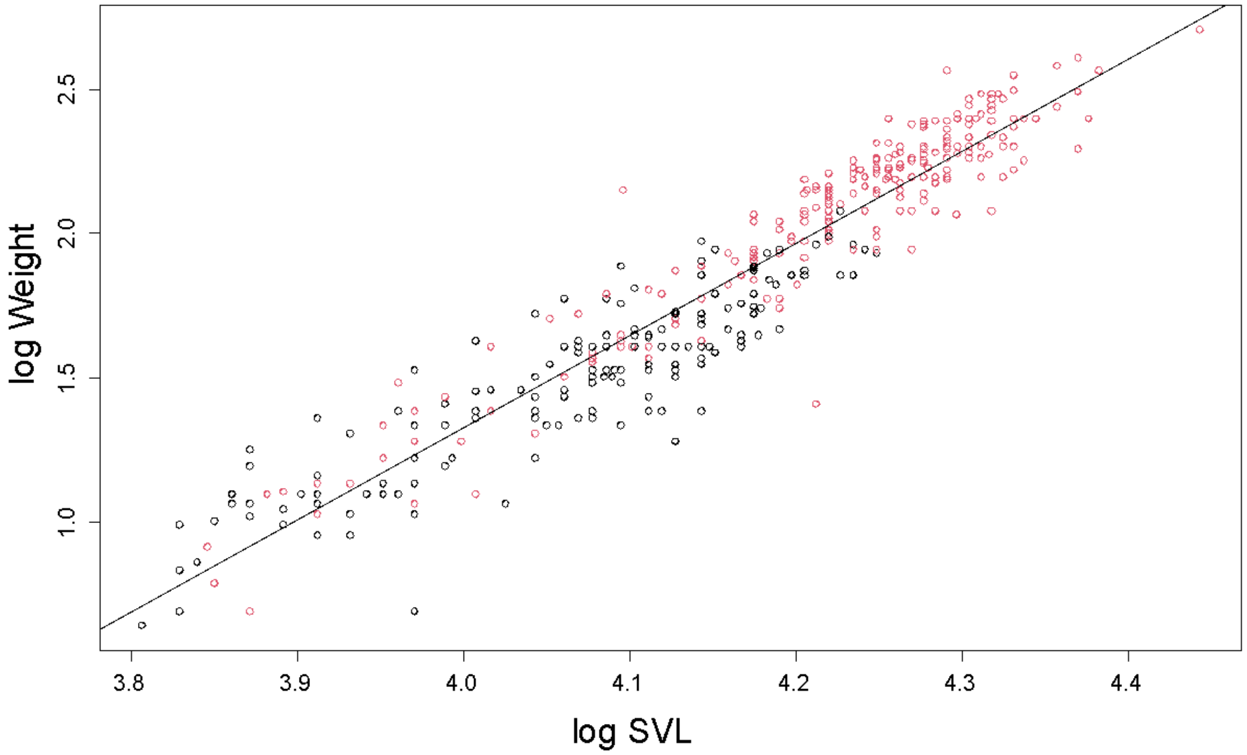

Figure S3. Regression plot of weight on SVL. The residuals of this model were used as body condition index. Male: red dots, female: black dots.

Table S1. Sample size of *Podarcis siculus* populations. Data: SVL = snout-vent length, and GM = geometric morphometrics. The individuals were collected in the seven main islands of the Aeolian archipelago and in the three islets around Panarea. M = males, F = females, TOT = total

Island	SVL_M	SVL_F	SVL_{TOT}	GM_M	GM_F	GM_{TOT}
Alicudi	33	22	55	22	13	35
Filicudi	27	21	48	21	8	29
Salina	34	21	55	26	16	42
Panarea	35	24	59	33	24	57
Stromboli	35	24	59	21	10	31
Lipari	26	21	47	21	11	32
Vulcano	32	24	56	29	18	47
**Islet**	SVL_M	SVL_F	SVL_{TOT}	GM_M	GM_F	GM_{TOT}
Bottaro	5	2	7	5	2	7
Lisca Bianca	6	6	12	6	6	12
Basiluzzo	6	4	10	6	4	10
TOT	239	169	408	190	112	302

Table S2. Candidate models assessing the effect of environmental predictors on phenotypic differences between females and males at different scales: a) individual, and b) island. The dependent variables of models are: snout-vent length (SVL); head shape; body size dimorphism, and head shape dimorphism. Models are ranked according to their AICc values. ΔAICc = difference between the AICc of a model and the best AICc; *w* = Akaike's weight of the model; R^2_M and R^2_C marginal and conditional R^2 respectively. The sign of the relationship is in parentheses. BCI = body condition index, NDVI = Normalized Difference Vegetation Index, Sex = sex of the lizards, Temp = land surface temperature.

a) Individual-level analysis

**Dependent**	**Predictors**	**AICc**	**Δ AICc**	***w***	R^2_M	R^2_C
SVL	BCI (-), NDVI (+), Sex, Sex*BCI, Sex*NDVI	-797.44	-	>0.99	0.51	0.55
	BCI (-), NDVI (+), Temp (-), Sex, Sex*BCI, Sex*NDVI, Sex*Temp	-793.32	4.12	-	-	-
	BCI (-), Sex*BCI	-774.57	22.87	≤0.02	-	-
	BCI (-), Temp (-), Sex, Sex*BCI, Sex*Temp	-772.13	25.31	-	-	-
	NDVI (+), Sex, Sex*NDVI	-693.55	103.90	≤0.02	-	-
	NDVI (+), Temp (-), Sex, Sex*NDVI, Sex*Temp	-692.47	104.97	-	-	-
	Sex	-675.51	121.94	-	-	-
	Temp (-), Sex, Sex*Temp	-675.24	122.21	-	-	-
Head shape	BCI (-), NDVI (+), Sex, Sex*BCI, Sex*NDVI	-1618.57	-	0.36	0.44	0.49
	BCI (-), Sex, Sex*BCI	-1618.40	0.17	0.33	0.42	0.49
	BCI (-), NDVI (+), Temp (+), Sex, Sex*BCI, Sex*NDVI, Sex*Temp	-1618.39	0.18	-	-	-
	NDVI (+), Temp (+), Sex, Sex*NDVI, Sex*Temp	-1617.44	1.13	0.20	0.44	0.48
	BCI (-), Temp (+), Sex, Sex*BCI, Sex*Temp	-1616.82	1.75	-	-	-
	NDVI (+), Sex, Sex*NDVI	-1616.21	2.36	≤0.02	-	-
	Sex	-1615.75	2.82	-	-	-
	Temp (+), Sex, Sex*Temp	-1615.36	3.21	-	-	-

b) Island-level analysis

**Dependent**	**Predictors**	**AICc**	**Δ AICc**	***w***	R^2	R^2_C
Body size dimorphism	BCI (+), NDVI (+)	-30.9	-	0.45	0.68	-
	BCI (+)	-30.8	0.1	0.43	0.48	-
	NDVI (+)	-28.2	2.7	0.12	0.34	-
	-	-27.89	2.99	-	-	-
	Temp (+)	-26.49	4.38	-	-	-
	BCI (+), Temp (+)	-26.19	4.68	-	-	-
	NDVI (+), Temp (+)	-22.98	7.89	-	-	-
	BCI (+), NDVI (+), Temp (+)	-21.90	8.97	-	-	-
Head shape dimorphism	BCI (+)	-67.60	-	>0.99	0.43	-
	-	-65.81	1.79	-	-	-
	Temp (+)	-65.04	2.57	-	-	-
	BCI (+), Temp (+)	-63.78	3.83	-	-	-
	NDVI (+)	-62.71	4.89	-	-	-
	BCI (+), NDVI (+)	-62.06	5.54	-	-	-
	NDVI (+), Temp (+)	-59.05	8.55	-	-	-
	BCI (+), NDVI (+), Temp (+)	-54.78	12.82	-	-	-

Table S3. The best-AICc models after removing three islets (Bottaro, Basiluzzo, Lisca Bianca) showed smaller sample size than larger islands, at different scales: a) individual, and b) island. The dependent variables of models are: snout-vent length (SVL); head shape; body size dimorphism, and head shape dimorphism. BCI = body condition index, NDVI = Normalized Difference Vegetation Index, Sex = sex of the lizards.

**a) Individual scale**	**Sex*BCI**			**Sex*NDVI**			**Sex**		
	**df**	**F**	***p***	**df**	**F**	***p***	**df**	**F**	***p***
SVL	1, 378.24	111.659	<0.001	1, 373.9	3.493	0.062	1, 373.81	5.89	0.016
Head shape	1, 267.17	4.393	0.037	1, 265.68	1.579	0.21	1, 265.77	4.565	0.034

**b) Island scale**	**BCI**			**NDVI**		
	**df**	**F**	***p***	**df**	**F**	***p***
Body size SD	1, 4	7.199	0.055	1, 4	6.257	0.067
Head shape SD	1, 5	11.772	0.019	-	-	-

Table S4. The best-AICc models after replacing ecosystem productivity (NDVI = Normalized Difference Vegetation) with island area, at different scales: a) individual, and b) island. The dependent variables of models are: snout-vent length (SVL); head shape; body size dimorphism, and head shape dimorphism. BCI = body condition index, area = island area, Sex = sex of the lizards.

**A) Individual scale**	**Sex*BCI**			**Sex*Area**			**Sex**		
	**df**	**F**	***p***	**df**	**F**	***p***	**df**	**F**	***p***
SVL	1, 406.35	117.118	<0.001	1, 399.17	0.808	0.369	1, 398.68	270.238	<0.001
Head shape	1, 295.678	4.272	0.04	1, 293.232	1.795	0.181	1, 293.172	240.184	<0.001

**B) Island scale**	**BCI**			**Area**		
	**df**	**F**	***p***	**df**	**F**	***p***
Body size SD	1, 7	12.843	0.009	1, 7	5.227	0.056
Head shape SD	1, 7	11.772	0.019	-	-	-
